# The relationship between internet-gaming experience and executive functions measured by virtual environment compared with conventional laboratory multitasks

**DOI:** 10.1371/journal.pone.0198339

**Published:** 2018-06-07

**Authors:** Yong-Quan Chen, Shulan Hsieh

**Affiliations:** 1 Department of Psychology, National Cheng Kung University, Tainan, Taiwan; 2 Institute of Allied Health Sciences, National Cheng Kung University, Tainan, Taiwan; Ariel University, ISRAEL

## Abstract

The aim of this study was to investigate if individuals with frequent internet gaming (IG) experience exhibited better or worse multitasking ability compared with those with infrequent IG experience. The individuals’ multitasking abilities were measured using virtual environment multitasks, such as Edinburgh Virtual Errands Test (EVET), and conventional laboratory multitasks, such as the dual task and task switching. Seventy-two young healthy college students participated in this study. They were split into two groups based on the time spent on playing online games, as evaluated using the Internet Use Questionnaire. Each participant performed EVET, dual-task, and task-switching paradigms on a computer. The current results showed that the frequent IG group performed better on EVET compared with the infrequent IG group, but their performance on the dual-task and task-switching paradigms did not differ significantly. The results suggest that the frequent IG group exhibited better multitasking efficacy if measured using a more ecologically valid task, but not when measured using a conventional laboratory multitasking task. The differences in terms of the subcomponents of executive function measured by these task paradigms were discussed. The current results show the importance of the task effect while evaluating frequent internet gamers’ multitasking ability.

## Introduction

This era of digital technology has brought about revolutionary changes to human civilization. With the development of digital technology, people tend to perform multiple tasks at the same time more often to make their lives more productive. In this study, we are particularly interested in exploring whether more frequent internet gaming (IG) is associated with higher or lower efficacy in multitasking ability. Additionally, we also compare the multitasking ability measured using a naturalistic-based task (i.e., in a virtual environment) with that measured using conventional laboratory executive function tasks.

There is no consensus in previous research related to these research questions. For example, Dong et al. [1] examined participants with internet addiction, as measured using the internet addiction test (IAT) [2], and observed that they had lower cognitive flexibility according to the conventional laboratory-based Stroop task [3]. Several other studies also observed poorer cognitive function, especially cognitive control, which is associated with frequent internet use [4–10].However, Boot et al. [11] observed that video game experts outperformed non-gamers in many cognitive tasks, including tracking moving objects at greater speeds, performing more accurately in a visual short-term memory test, switching between tasks more quickly, and making decisions about rotated objects more quickly and accurately. Several other studies also observed a positive effect of internet use related to various cognitive functions, including visual and visuospatial attention, dual tasks, inhibition, planning, working memory, episodic verbal memory, visual and verbal reasoning, and fluid intelligence [8, 12–25].

Previous studies have mostly used conventional laboratory tasks that have been challenged in terms of their representativeness of an individual’s abilities in accomplishing real-life activities. For example, in the memory research domain, evidence has shown that using conventional laboratory tasks would affect the evaluation outcome regarding an individual’s cognitive efficacy compared with naturalistic tasks in real or virtual environment. In another example, conventional laboratory prospective memory tasks are related to memory for future intentions. Participants could be asked to perform an ongoing task and occasionally respond to prospective memory cues to perform another expected task, which is a form of dual tasks [26–27].

Researchers have observed that older adults are impaired in conventional prospective memory tasks, but they seem to outperform younger adults on naturalistic prospective memory tasks, such as remembering to make phone calls or to take medications, even when the participants are asked not to use external aids [28]. Along these lines, we speculated that previous studies investigating the issue of whether frequent internet use is associated with higher or lower cognitive efficacy might have overlooked the effect of conventional laboratory task paradigms compared to naturalistic paradigms. Therefore, we compared multitasking abilities between two groups of participants with frequent or infrequent internet-gaming experience using conventional laboratory and virtual environment task paradigms.

In a laboratory setting, researchers have developed representative tasks to measure multitasking abilities, such as dual-task [29–31] and task-switching [32–34] paradigms. Logie developed the Edinburgh Virtual Errands Test (EVET) to mimic everyday multitasking activities in a virtual environment. In the conventional laboratory dual-task paradigm, participants are usually required to perform two tasks in parallel, whereas in the EVET, participants perform a series of tasks in a particular order and by interleaving, or switching from one to the other, when each of the tasks is completed [35]. In the conventional laboratory task-switching paradigm, rapid task switching is required, whereas tasks in the EVET involve much longer time scales where rapid and accurate response times are less crucial, and most of the tasks have a clear end point [35].

The performance on the EVET has been suggested to be a good predictor of problems with planning and “intentionality” (i.e., goal-directed behavior) in everyday life [36]. Studies have also shown that the virtual environment of the EVET can reflect task performance in a real-life environment [37]. The EVET involves a wide range of cognitive functions acting in concert, such as retrospective and prospective memory, working memory, planning, and implementation of a plan to achieve multiple goals, such as completing multiple errands as instructed. In contrast, the majority of tasks in a laboratory setting focus on individual cognitive functions in relative isolation and give less attention to various cognitive abilities simultaneously [35].

However, coordinating and strategically deploying several different cognitive functions are essential in everyday life scenarios, especially in a digital technology environment. In other words, everyday multitasking is different from concurrent dual-task demands [29–31] or from the microstructure of rapid switching between laboratory tasks [32–34]. Instead, it involves several subtasks that have different requirements and how these subtask attempts are scheduled by individuals [35]. The conventional laboratory dual-task and task-switching paradigms address only specific components of the cognitive system [35], such as the core (or “primary-order”) subcomponents of working memory and cognitive flexibility, respectively in the Diamond’s executive function model [38]

Therefore, we explored these different aspects of cognitive processes that may be involved in conventional laboratory or virtual environment task paradigms. We hypothesized that frequent internet gamers might not necessarily perform similarly between a conventional laboratory task and a virtual environment task. Additionally, the frequent IG group might not necessarily perform more poorly than infrequent IG group when evaluated using a more naturalistic task such as the EVET.

## Materials and methods

This study protocol was approved by the Human Research Ethics Committee of the National Cheng Kung University, Tainan, Taiwan, R.O.C. to protect the participants’ right according to the Declaration of Helsinki and the rule of research at the University. All participants signed an informed consent form before participating in the experiments.

## Participants

Seventy-eight young healthy college students who were recruited via a bulletin board system and advertisements in BBS and on Facebook participated in this study. Two of them did not fill in questionnaires properly and 4 of them did not complete the experimental tasks, hence only 72 (36 females; 36 males) participants’ data remained to be analyzed. The sample size was chosen based on prior research that analyzed the lowest and highest 25% of media-use scores from 92 participants, resulting in 23 participants for each group [12]. The two groups’ switch costs reached statistical significance at p = 0.02. In order to reach a power of 0.8 and p < 0.05, the estimated sample size was at least 18 subjects per group. The remaining participants were right-handed, and 20 to 30 years old (mean age, 23.43; SD, 2.21). Individuals aged from 20 to 30 years are considered as digital natives (https://en.wikipedia.org/wiki/Digital_native). No history of neurological, psychiatric disorders, cardiovascular diseases or any forms of addiction (e.g., alcohol, drug, internet, etc.) was detected in the self-report. All participants were not at risk of depression or anxiety disorder, as evaluated using the Beck Depression Inventory II (scores < 13; [39–41]) and Beck Anxiety Inventory (scores < 7; [42–43]). Each participant received an honorarium of NT$350 (US $12) for completing the study.

### Experimental instruments

#### Virtual environment multitasking task: Edinburgh Virtual Errands Test

This study used the EVET [44–46](http://www.psy.ed.ac.uk/resgroup/MT/index4.html) to evaluate participants’ multitasking ability in a virtual naturalistic setting. EVET requires participants to complete eight errand tasks efficiently within 8 minutes while navigating through a simulated environment on a computer. The environment consisted of a four-story building with a set of stairs and five rooms along the left and right ends of each floor surrounding a central elevator.

Each participant was provided with instructions as follows before receiving the experimental test: “Please imagine that you are a student and are assigned to do a list of errands for your teacher. The errands are listed in a particular order, but you can vary the order at any time as you wish. However, you are also told not to enter any of those rooms unless the rooms are on the list. You have eight minutes to complete these assignments. Please complete all the assignments as soon as you can, and complete as many as possible.”

An example of the errand list is as follows:

- Pick up the brown package in T4 and take it to G6
- Pick up the newspaper in G3 and take it to the Desk in S4
- Obtain the keycard in F9 and unlock G6 (via G5)
- Meet the person in S10 before 3:00 min
- Obtain the stair-code from the notice board in G8 and unlock the stairwell
- Turn on Cinema S7 at 5:30 min
- Turn off the Lift G Floor
- Sort the red and blue binders in room S2. Sort as many binders as you can.

G = ground floor; F = first floor; S = second floor; T = third floor

**Fig 1:**
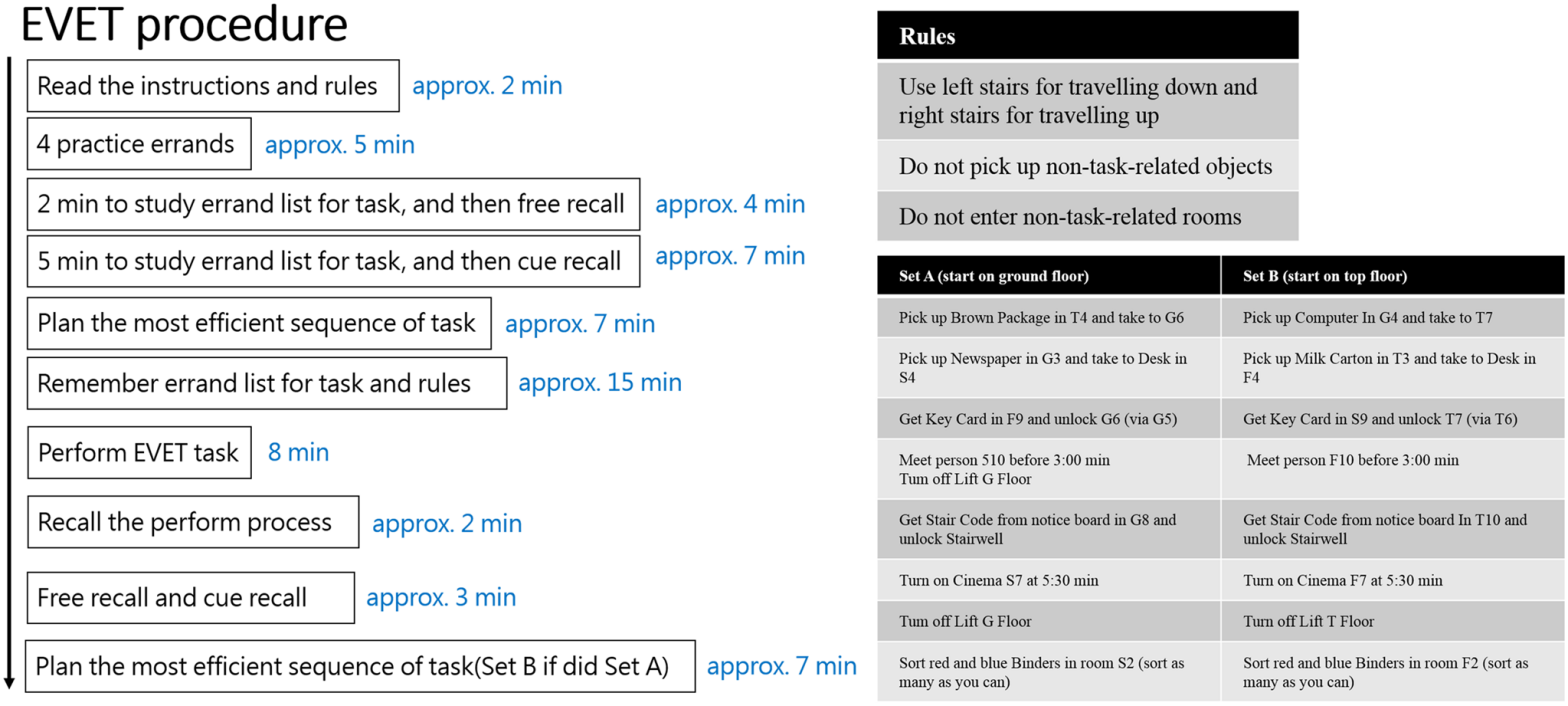
**A. Pipeline of Edinburgh Virtual Errands Test (EVET) experiment.** Participants familiarized themselves with the building plan and task errands and performed the EVET. B. Task rules and two sets of errands.

In the EVET experiment, participants were given 2 minutes to study their errand list, followed by free recall, then another 5 minutes of further study, and a test of cued recall. Then, participants were asked to plan the order in which they should perform each errand to achieve the maximum efficiency in task completion. Next, they were asked to verbally recall the errand list and building rules until they could recall 100% of the list. Participants then performed the EVET (i.e., navigating through a simulated environment) for 8 minutes (neither errand list nor plan were present during the navigation test). Afterwards, they were asked to recall the errand they had attempted or failed to complete. Participants were cued about any errands they had omitted, along with the errands correctly recalled from the recount task. Finally, participants were given an alternative set of errands (Set A or Set B; see Fig 1) and were asked to plan the order of errands (e.g., 35 participants who performed set A were subsequently asked to plan set B, and vice versa), which provided another measure of planning, but without performing the EVET a second time.

A general “EVET score” was calculated based on participants’ overall performance (accounting for completed errands and incorrect actions). Points were added for each errand completion, bonus points were awarded based on the number of folders sorted and the time discrepancy for timed errands, and points were deducted for picking up incorrect objects, entering rooms not on the errand list, and breaking the building rules. Bonus and penalty points were given/deducted on a five-point scale (0–4) based on a cutoff score that was calculated based on the frequency distribution of raw scores among the participant sample in Logie et al.’s study (2011)[44]. The minimum possible score was −12 and the maximum was 20. The EVET subscores were calculated as follows:

**Travel time:** the total time (in seconds) during which the participant was navigating within the virtual building minus the time spent on entering the room. The purpose was to calculate the participants’ implementation of the mission navigation efficiency.

**Learn:** the sum of the free and cued recall scores. Before participants performed the EVET test, they were requested to recall (free and cued recall) the errands. If they could recall a key point, they received 1 point. The maximum number of points for the errands was 42.

**Recount:** After the EVET test, participants were immediately asked to recall any violations of the rules that occurred during the EVET test. If participants could recall a key word, they received 1 point. The maximum possible score was 28.

**Remember:** the sum of the free and cued recall scores. After participants performed the EVET test, they were requested to recall (free and cued recall) the errands. If they could recall one key point they received 1 point toward their score. The maximum number of points was 42 for the errands.

**Pretest and posttest plans:** the plan score before and after the EVET test. Both pretest and posttest plans were compared to the averaged plan of the top three participants’ EVET scores, respectively.

**Plan follow:** the EVET pretest plan was compared with the actual completion order of the EVET test. If the planning and the actual completion order’s locations/positions were the same, the participant received 1 point. For example, if the planning order was ABC, and the actual completion order was BCA, in which C was completed after B, the participant received 1 point.

### Conventional laboratory multitasking task

#### Dual task

The dual task was adapted from the website of *Cognition Laboratory Experiments*, designed by John H. Krantz (https://psych.hanover.edu/JavaTest/CLE/Cognition/Cognition/dualtask_instructions.html). In this version of a dual-task experiment, there was a primary visual tracking task where participants tried to keep a dot inside of a rectangle box, similar to following the road. During this tracking task, letters (or sounds) might appear and if a target letter or an auditory sound appeared, participants had to respond to it by clicking the mouse (i.e., a dual-task condition in which the secondary task could be a visual or an auditory task).

**Fig 2:**
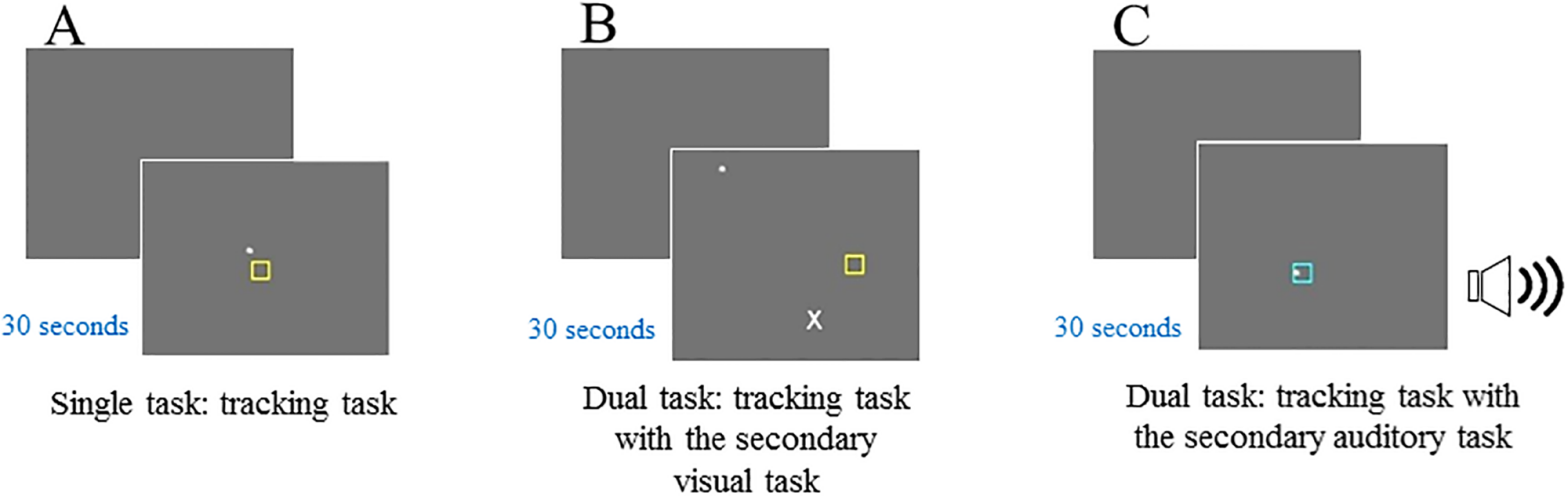
An example of a dual task. A: A single-task condition: the primary tracking task when the dot was outside of the box. B: A dual-task condition: the primary tracking task with the secondary visual (letter “X”) task. C: A dual-task condition: the primary tracking task with the secondary auditory (sound 1000 Hz) task.

During the single-task condition, if participants successfully maintained a dot inside the rectangle box, then the box would turn to a cyan color and no tracking error would be recorded (Fig 2). Participants were encouraged to keep the box the cyan color by maintaining the dot inside the box. The range for the direction of the dot’s motion to be tracked could change from 0 to 360 degrees with each update. The smaller the angle variation, the more direct was the movement of the dot, and thus it was easier to follow. The larger the angle variation, the more random was the movement of the dot, and thus it was more difficult to follow. The speed of the dot was eight pixels per update and the diameter of the dot was four pixels. In the dual-task condition (visual–visual or visual–auditory task), while participants were tracking the dot, a letter (i.e., a visual secondary task) or an auditory sound (i.e., an auditory secondary task) might appear (Fig 2). If a target letter (“X”) or sound (1000 Hz) appeared (for the duration of 150 ms followed by a stimulus-onset asynchrony of 500 ms), participants had to respond to it by clicking the left button of the mouse. When other non-target letters or sounds appeared, no responses were needed. There were a total of two single-task and two dual-task blocks (one block with a visual task as the secondary task, and one block with an auditory task as the secondary task), consisting of 15 trials per block.

The dual-task costs for the primary tracking task were measured by calculating the number of pixels between the box and the dot (the smaller the numbers of pixels, the better is the performance) in single-task and dual-task conditions respectively, and then the pixels in the single-task condition were subtracted from those in the dual-task condition. There were two types of dual-task costs: one visual dual-task cost (with a letter task as the secondary task) and one auditory dual-task cost (with an auditory task as the secondary task).

#### Task-switching paradigm

**Fig 3:**
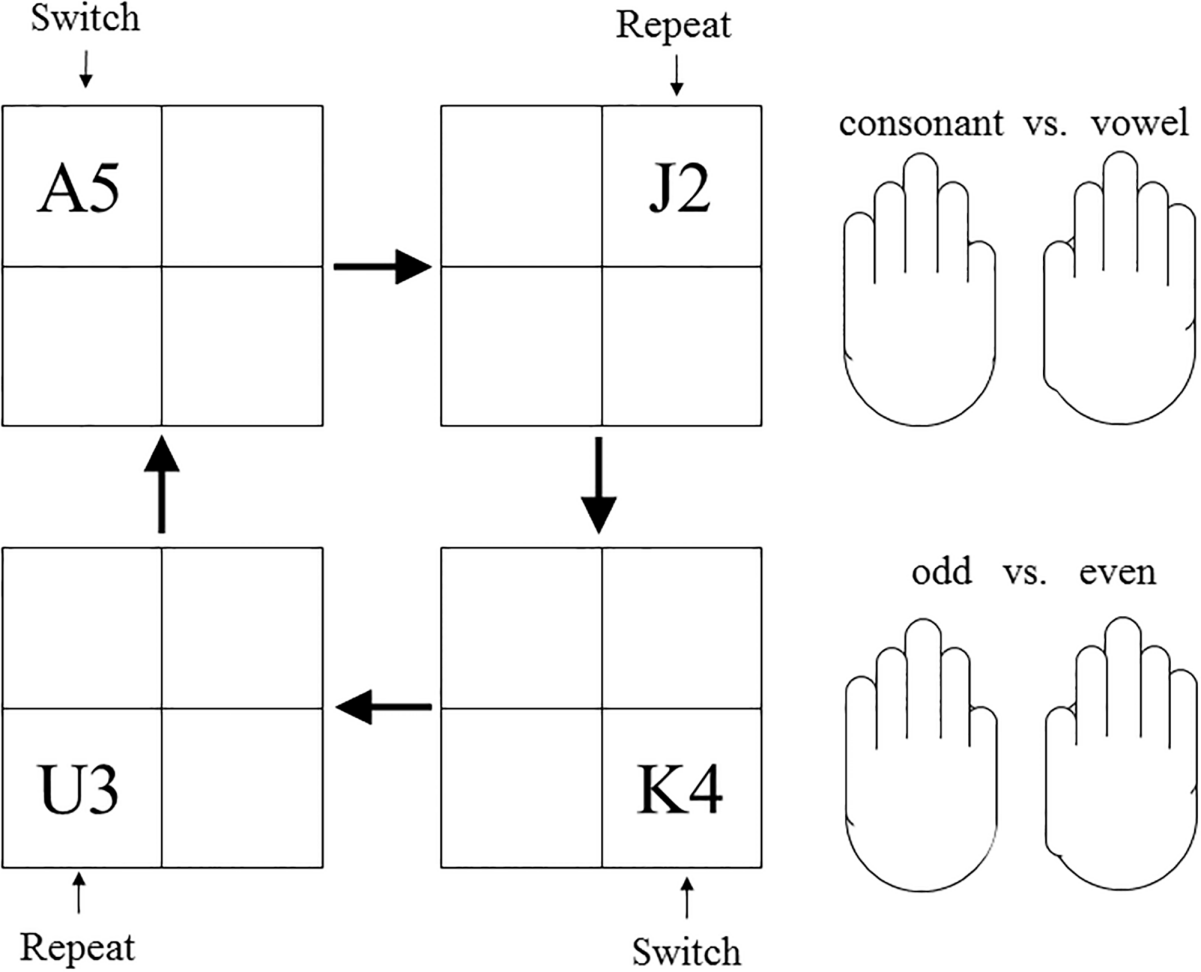
An example of task switching. Stimuli that appear in the upper two positions indicate Task A (e.g., letter task; consonant vs. vowel judgment task), where in the lower two positions indicate Task B (e.g., digit task: odd vs. even number task). Participants were instructed to respond to consonants or odd numbers by pressing the key with their left finger, and to vowels or even numbers by pressing the key with their right finger (stimulus–response mapping was counterbalanced across participants).

The task-switching paradigm was adapted from the alternating-runs procedure paradigm designed by Rogers and Monsell (1995; Experiment 1)[34]. There were four consonants (G, K, M, R) and four vowels (A, E, I, U) as the letter task’s stimuli, and four odd numbers (3, 5, 7, 9) and four even numbers (2, 4, 6, 8) as the digit task’s stimuli. In the alternating-run (mixed-task) blocks, participants alternated between runs of two trials presented in one of four quadrants (i.e., the spatial version, see [34]). For example, stimuli that appear in the upper two positions indicate Task A (e.g., letter task; consonant vs. vowel judgment task), where in the lower two positions indicate Task B (e.g., digit task: odd vs. even number task; Fig 3). Participants were instructed to respond to consonants or odd numbers by pressing the key with their right finger, and to vowels or even numbers by pressing the key with their left finger (stimulus-response mapping was counterbalanced across participants).

Rogers and Monsell (1995)[34] computed switch costs by subtracting the average reaction time (RT) or accuracy of the repeat trials (the second trial on the sequence of Task A–Task A and Task B–Task B) from those of the corresponding switch trials (the second trial on the sequence of Task A–Task B and Task B–Task A). In this way, switch costs (also known as ***local switch costs***) can be measured accurately without contamination from unwanted differences in factors such as memory load, effort, or arousal (i.e., ***mixing costs***). However, in the pure (single)-task blocks, participants performed the same task (either a letter task or digit task) throughout all quadrant positions. The mixing cost can be measured by subtracting the average RT or accuracy of the repeated trials in the pure-task blocks from those of the repeated trials in the mixed-task blocks. There were a total of two single-task blocks (one for the letter task; one for the digit task), and two alternating-run task blocks, consisting of 65 trials per block.

### Procedure

Each participant first provided written informed consent, and was then given the BDI-II, BAI, Chen’s Internet Addiction Scale (CIAS; [47]), and Internet Use Questionnaire [48] to complete. The Internet Use Questionnaire was used to evaluate the amount of experience each participant had in playing online games. The participants were split into groups based on the following questions: Do you play any online game (tablet, computer or mobile phone) with interaction with other players? How many hours do you spend on playing such online game? Do you play any online game with a single operation? How many hours?

After the questionnaires were completed, each participant performed the EVET, dual-task, and task-switching paradigms on the computer. All of the experiments were completed in an average of about 2.5 hours.

### Statistical analysis

All the statistics were performed using R software (R x64 3.4.0), and two-tailed Welch’s unequal variances *t*-tests were used. The significance level was set at 0.05 (uncorrected). The effect size and power tests were performed using the power analysis package offered by R software (https://cran.r-project.org/web/packages/powerAnalysis/powerAnalysis.pdf). Spearman’s ρ correlation analysis was used to explore the associations among EVET scores, dual-task cost, switch cost, mixing cost, time spent on using the internet and playing online games, and CIAS.

## Results

Minimal data set for Tables 1–5 presented in the Results section can be found in S1 File.

**Table 1:** Classification of frequent vs. infrequent internet-gaming (IG) experience groups based on the questions regarding the hours playing online game types (playing with others or alone) respectively in the Internet Use Questionnaire (Lin, 2011). These groups’ demographic information and their Chen Internet Addiction Scale (CIAS) scores were also compared.

Internet Game Type	Frequent IG experience group	Infrequent IG experience group	*t*(df) value	*p value*	range
Age	23.33 (2.25)	23.52 (2.19)	t(69.94) = -0.37	.72	20~30
Education	16.25 (1.38)	16.53 (1.30)	t(69.73) = -0.88	.38	13~20
BDI	4.19 (3.31)	3.67 (3.42)	t(69.92) = 0.67	.51	0~13
BAI	1.66 (1.74)	1.97 (1.99)	t(69.75) = -0.69	.49	1~7
Time spent on online interaction game playing (hours)	8.72 (7.10)	0.06 (0.23)	t(35.08) = 7.3	1.47e-08	0~28
Time spent on online single-operation game playing (hours)	3.97 (5.33)	0.54 (1.41)	t(39.89) = 3.74	.001	0~21
CIAS score	58.17 (11.17)	53.28 (13.22)	t(68.10) = 1.70	.09	27~86

*Note*: All data are represented as mean (standard deviation). BDI: Beck’s Depression Inventory; BAI: Beck’s Anxiety Inventory; IG: internet-gaming; CIAS: Chen’s Internet Addiction Scale; df: degree of freedom

**Table 2:** Statistical tests between the EVET total score and sub-test scores between Set A and Set B.

	Set A	Set B	*t*(df) value	*p value*	range
EVET score	9.17 (4.93)	11.64 (5.37)	t(69.49) = -2.03	.054	0~19
EVET travel time (sec)	323.92 (38.60)	304.58 (58.73)	t(60.49) = 1.65	.10	178~413
EVET learn	22.75 (6.26)	21.86 (6.34)	t(69.99) = 0.60	.55	7~41
EVET recount	14.97 (5.14)	16.75 (4.98)	t(69.93) = -1.49	.14	4~24
EVET posttest plan	5.81 (1.88)	4.86 (2.22)	t(68.16) = 1.95	.06	2~11
EVET plan follow	5.19 (2.57)	6.14 (3.02)	t(68.30) = -1.43	.16	1~11

*Note*: All data are represented as mean (standard deviation). EVET: Edinburgh Virtual Errands Test; sec: seconds; df: degree of freedom

**Table 3:** Statistical tests for the EVET total score and subtest scores between the groups of frequent internet-gaming (IG) experience and infrequent IG experience.

	Frequent IG experience	Infrequent IG experience	*t*(df) value	*p value*	Effect size	power
EVET score (%)	65.70 (22.75)	38.35 (22.49)	t(69.99) = 5.13	2.45e-06	1.21	.999
EVET travel time (sec)	295.14 (53.48)	331.36 (38.99)	t(64.01) = -3.47	.001	0.82	.93
EVET learn (%)	53.98 (12.62)	52.24 (17.07)	t(64.46) = 0.49	.63	0.11	.08
EVET recount (%)	63.29 (15.05)	50.00 (18.90)	t(66.65) = 3.30	.002	0.78	.90
EVET remember (%)	87.36 (10.10)	86.98 (8.10)	t(66.84) = 0.18	.86	0.04	.05
EVET pretest plan (%)	55.09 (19.19)	40.91 (16.19)	t(68.07) = 3.38	.001	0.80	.92
EVET posttest plan (%)	52.00 (21.25)	44.91 (16.11)	t(65.24) = 1.59	.12	0.38	.35
EVET plan follow (%)	64.18 (25.24)	38.91 (19.27)	t(65.45) = 4.77	1.07e-05	1.12	.997

*Note*: All data are represented as mean (standard deviation). IG: internet-gaming; sec: Seconds; EVET: Edinburgh Virtual Errands Test; df: degree of freedom

**Table 4:** Statistical results for dual task performance (dual-task cost) and task switching (switch cost; mixing cost) between the groups of frequent internet-gaming experience and infrequent internet-gaming experience.

Dual task and task switching items	Frequent IG experience group	Infrequent IG experience group	*t*(df) value	*p value*	Effect size	Power
Dual-task cost (visual)	2.07 (1.87)	4.80 (8.36)	t(38.51) = -1.91	.06	0.45	.46
Dual-task cost (auditory)	1.03 (2.31)	3.21 (9.72)	t(38.95) = -1.31	.20	0.31	.25
Switch cost (RT; ms)	352.88 (248.47)	395.31 (179.78)	t(63.76) = -0.83	.41	0.20	.13
Mixing cost (RT; ms)	110.25 (115.16)	155.06 (151.94)	t(65.23) = -1.41	.16	0.33	.29
Switch cost (ACC; %)	2.52 (3.09)	2.52 (4.67)	t(60.73) = 0.01	.99	0.002	.05
Mixing cost (ACC; %)	-0.35 (3.31)	-1.79 (3.56)	t(69.63) = 1.77	.08	0.37	.34
Dual-task tracking	13.31 (1.53)	13.27 (1.72)	t(69.04) = 0.11	.92	0.03	.05
Dual-task tracking (visual)	15.38 (2.35)	18.06 (9.37)	t(39.41) = -1.67	.10	0.39	.38
Dual-task tracking (auditory)	14.34 (3.28)	16.48 (10.56)	t(41.69) = -1.16	.25	0.27	.21
pure repeat RT (ms)	714.67 (121.79)	723.03 (93.22)	t(65.53) = -0.33	.74	0.08	.06
repeat trial RT (ms)	824.86 (171.37)	878.08 (184.37)	t(69.63) = -1.27	.21	0.30	.24
switch trial RT (ms)	1177.78 (343.45)	1273.33 (229.02)	t(60.99) = -1.39	.17	0.33	.28
repeat trial ACC (%)	97.40 (2.77)	97.27 (2.49)	t(69.21) = 0.21	.84	0.05	.05
switch trial ACC (%)	94.88 (3.19)	94.75 (4.83)	t(60.61) = 0.13	.90	0.03	.05

*Note*: All data are represented as mean (standard deviation). IG: internet-gaming; RT: reaction time; ms: milliseconds; ACC: Accuracy; %: percentage; df: degree of freedom

**Table 5:** Correlation matrix among EVET, dual-task cost, switch cost, mixing cost, time spent on online game and Chen Internet Addiction Scale (CIAS) score.

	Mean	*SD*	1	2	3	4	5	6	7	8	9	10	11	12	13	14	15	16	17
1. EVET score (%)	0.52	0.26																	
2. EVET travel time (s)	314.25	50.29	-.67**																
3. EVET learn (%)	0.53	0.15	.37**	-.39**															
4. EVET recount (%)	0.57	0.18	.53**	-.27*	.32**														
5. EVET remember (%)	0.87	0.09	.17	-.14	.44**	.40**													
6. EVET pretest plan (%)	0.48	0.19	.46**	-.41**	.03	.32**	.02												
7. EVET posttest plan (%)	0.48	0.19	.16	-.16	.03	.15	-.23*	.17											
8. EVET plan follow (%)	0.52	0.26	.58**	-.45**	.20	.66**	.25*	.48**	.25*										
9. Dual-task cost (visual)	3.43	6.17	-.23*	.25*	-.25*	-.09	-.01	.002	-.05	-.09									
10. Dual-task cost (auditory)	2.12	7.1	-.12	.18	-.09	-.02	-.03	.08	.04	-.02	.91**								
11. Switch cost (RT) (ms)	374.09	216.38	-.14	.14	-.16	-.02	-.12	.03	.21	-.11	.05	.06							
12. Mixing cost (RT) (ms)	132.65	135.75	-.27*	.20	-.28*	-.21	-.09	-.15	-.11	-.19	.10	-.02	-.18						
13. Switch cost (ACC)	2.52	3.93	.07	.002	-.04	.02	-.06	-.05	.07	.1	-.03	-.04	-.08	.18					
14. Mixing cost (ACC)	-1.07	3.49	.06	-.03	.03	.06	.14	.12	.01	.18	-.13	-.08	.09	-.16	-.52**				
15. CIAS score	55.72	12.4	.2	-.15	.13	.23	.19	.09	-.04	.15	-.14	-.14	.10	-.04	-.17	.09			
16. Time spent on online interaction game playing (h)	4.39	6.63	.34**	-.21	.04	.17	.01	.08	.004	.24*	-.16	-.13	-.06	-.05	-.03	.04	.21		
17. Time spent on online single-operation game playing (h)	2.26	4.24	.36**	-.39**	.31**	.31**	.20	.26*	.10	.31**	-.10	-.09	-.18	-.11	.13	.02	.02	.20	
18. Time spent on internet use (h)	31.11	18.37	.18	-.20	.21	.10	0.14	.18	.09	.14	-.16	-.07	-.16	.004	.13	.11	.21	.30*	.28*

*Note*: EVET: Edinburgh Virtual Errands Test; sec: Seconds; RT: Reaction time; ms: milliseconds; ACC: Accuracy; h: Hours; SD: standard deviation;

“*”: p-value < .05;

“**”: p-value < .01

### The Internet Use Questionnaire

There were 36 participants in the frequent IG experience group (playing online game interacting with others: 8.72 ± 7.10 hours; playing online games alone: 3.97 ± 5.33 hours), and 36 participants in the infrequent IG experience group (playing online game interacting with others: 0.06 ± 0.23 hours; playing online game alone: 0.54 ± 1.41 hours). Please note, there were original 38 participants in the frequent IG experience group and 40 participants in the infrequent IG experience group as mentioned in the Participant section, but 6 of them were removed from the data analyses due to their incomplete questionnaires or experiments. The frequent IG group showed a significantly higher number of playing hours compared with the infrequent IG group (interacting with others: *t* = 7.32, *p* = 1.47e-08; alone: *t* = 3.74, *p* < .001; Table 1). The two groups’ demographic information is provided in Table 1.

### Chen Internet Addiction Scale score

The mean CIAS score for the frequent IG group was 58.17 ± 11.17, and for infrequent IG group was 53.28 ± 13.22. No significant difference was found between CIAS scores in the two groups (*p* = .09; Table 1).

### EVET performance

#### Set A vs. Set B

The EVET scores ranged from 0 to 19 in our sample. Participants who performed task set A (mean, 9.17, SD, 4.93) performed worse than those who performed set B (mean, 11.64, SD, 5.37), but this result only just reached marginal significance (*t* = −2.03, *p* = 0.054)(Table 2). Because there was no significant difference in the total score between the two EVET sets, we merged the data from these two sets in the subsequent analyses.

#### Frequent vs. infrequent internet-gaming groups

The frequent IG experience group showed significantly lower scores compared with the infrequent IG experience group for the total EVET score (*t* = 5.13, *p* = 2.45e-06; effect size: 1.21; power: .99), EVET travel time (*t* = −3.47, *p* = .001; effect size: 0.82; power: .93), EVET recount (*t* = 3.30, *p* = .0002; effect size: 0.78; power: 0.90), EVET pretest plan (*t* = 3.38, *p* = .001; effect size: 0.80; power: 0.92), and EVET plan follow (*t* = 4.77, *p* = 1.07e-05; effect size: 1.12; power: 0.99; Table 3). The results suggested that the frequent IG group exhibited overall better EVET performance than the infrequent IG group. Please note, in the frequent IG group, their types of gaming experience, such as tablet, computer or mobile phone, were controlled while comparing their EVET performance with that of the infrequent IG group. Twenty-eight out of the 36 participants played computer games, whereas 8 out of the 36 participants played mobile phone or tablet games. These two subgroups did not show significant differences in their EVET, dual task and task switching performance.

### Dual-task performance

Overall dual-task performance was not different between the two groups, including single-task tracking and dual-task tracking performance (Table 4). Their dual-task costs for the visual–visual dual-task condition and for the visual–auditory dual-task condition were not significantly different (visual–visual: *p* = .06; visual–auditory: *p* = .20; Table 4).

### Task-switching performance

The two groups did not differ from each other in overall task-switching performance, including the reaction time (RT) in the pure block condition, repeat, and switch trials in the mixed-task block conditions (Table 4). Their switch costs did not differ significantly from each other in terms of RT (*p* = .41) and accuracy (*p* = .99), and their mixing costs also did not differ significantly from each other in terms of RT (*p* = .16) and accuracy (*p* = .08; Table 4).

### EVET performance related to dual-task cost, switch cost, mixing cost, and internet gaming time

To summarize, only EVET scores differed significantly between the frequent and infrequent IG experience groups. Dual-task cost, switch cost, and mixing cost were not different between the two groups. We further examined if these variables were correlated among each other. Table 5 shows the correlation matrix among EVET, dual-task cost, switch cost, mixing cost, time spent on online game, and CIAS score.

## Discussion

The aim of this study was to investigate whether individuals with frequent IG experience exhibited better or worse multitasking ability than those with infrequent IG experience, using virtual environment and conventional laboratory tasks. The idea of comparing the EVET with dual tasks or task switching may not be novel [35, 44], but it has not been explored in young, healthy populations of frequent and infrequent internet gamers. The results showed that participants in the frequent IG group performed better than those in the infrequent IG group according to the EVET. However, the performance of frequent IG group on the dual task (visual–visual; visual–auditory) and task switching (switch cost; mixing cost) did not differ significantly from that of infrequent IG group.

The results suggest that the frequent IG group exhibited better multitasking efficacy when measured using a more natural task, but not when measured using a conventional laboratory multitasking task. To the authors’ knowledge, these findings have not been reported before in a single study. Therefore, the current results show the importance of the task effect where evaluating frequent internet gamers’ cognitive ability. This might also explain why there are discrepant findings on whether frequent internet use is associated with better multitasking ability. A similar phenomenon can be seen in perspective memory research, where naturalistic-based and laboratory-based PM tasks were shown to be differentially sensitive in reflecting memory efficacy [26–28], as well as in the neuropsychological research domain, where a higher ecological multitasking test (such as multiple-errands test [36] or Virtual Errands Test [37]) was more sensitive to evaluate executive dysfunction in brain-damaged patients. The frequent internet gamers examined did not necessarily have internet addiction disorder or internet gaming disorder according to the Chen Internet Addiction Scale (CIAS). Nine participants had CIAS scores greater than 68. We excluded these nine participants from the analyses. The results remained the same as reported. Therefore, the observation of better multitasking ability in the frequent IG group was not associated with internet addiction. The current findings are limited to individuals who play internet games in a healthy manner and do not represent those with addictive behavior.

A possible explanation for the different results between task paradigms is that a virtual naturalistic-based task is superficially similar to internet gaming scenarios. To test this hypothesis, we divided internet games into two types: those involving a gaming scenario that is different from the EVET (e.g., a shooting game such as a multiplayer online battle arena), and those involving a game scenario that is similar to the EVET (e.g., spatial navigation in a virtual environment). If the main reason why the EVET was more sensitive to evaluating frequent or infrequent internet gamers’ multitasking ability was simply because of the superficial task features, then frequent internet gamers who play games that differ from the EVET would exhibit worse EVET scores than those who play games that are more similar to the EVET. However, the EVET in these two subgroups should not differ if the better multitasking ability of internet gamers in the EVET is not only the result of superficial task similarity. We observed no difference between the subgroups.

Another more critical possibility from a theoretical point of view is that compared to the dual-task and task-switching paradigms, the EVET measures more cognitive components, including planning and execution, sustained attention, prospective memory, verbal and visuospatial working memory, task switching, processing speed, cognitive bottlenecks, and sequential control [35, 44]. In the EVET, participants perform a series of tasks in a particular order and by interleaving or switching from one to the other when each of the tasks is completed [35, 44]. In addition, an individual needs to memorize an errand list and preplan the order of the multiple errands to complete all tasks within 8 minutes while navigating around a virtual environment. Therefore, the current findings may suggest that the experience of playing internet games is more closely related to the ability to coordinate and strategically deploy several different cognitive functions, but not directly related to a single cognitive function, as often measured by a conventional laboratory task.

This phenomenon may be associated with the era of digital technology and social changes that have fueled the rise of multitasking in more complex scenarios. When playing online games, players must not only follow the rules but also often need to switch their attention back and forth between tasks, which is multitasking. These task demands may not be fully covered by conventional laboratory task paradigms, but they are covered by a more naturalistic-based EVET task. This could also partly explain why there were discrepancies regarding whether internet use was positively or negatively associated with cognitive functions [1–25]. The results may partly depend on whether one measures multiple cognitive functions in concert or in isolation.

However, further consideration is needed before fully advocating the use of a virtual naturalistic-based EVET task. According to Diamond’s executive function model [38], executive function has three core subcomponents (inhibitory control, working memory, and cognitive flexibility) and three higher-order subcomponents (reasoning, problem-solving, and planning). Based on this model, the EVET appears to contain more higher-order subcomponents, which are similar to aspects of fluid intelligence including the ability to reason, problem solving, and seeing patterns or relationships among items [49]. These higher-order subcomponents are not measured by the dual-task or task-switching paradigms, which instead measure the core (or “primary-order”) subcomponents of working memory and cognitive flexibility, respectively [38]. Therefore, the current findings may also suggest that playing internet games is more closely related to aspects of higher-order executive function (such as reasoning, problem-solving, and planning) but not to primary-order executive function. This study did not extensively measure all types of core and higher-order subcomponents using various laboratory-based tasks. It is likely that other types of conventional laboratory tasks that directly measure the higher-order subcomponents are also able to differentiate the abilities of frequent versus infrequent internet gamers. Future research is needed to clarify the issue.

If the EVET can sufficiently evaluate an individual’s multitasking ability in relation to different degrees of internet gaming experience (see [50]), applying it in clinical assessments for intervention programs may be more convenient than using a combination of multiple conventional laboratory tasks. Video games have become popular and more available in the past several decades and they have become even more easily accessible through handheld devices such as mobile phones or tablets, which provide users with unlimited access. Moreover, there is growing interest in video game development for treating organic brain diseases in elderly patients [51–52] or attention deficit hyperactivity disorder (ADHD) [53]. Therefore, choosing a concise and sensitive task to evaluate the outcomes of intervention programs is important for clinical research.

Assessments and outcome measures have important roles in the effectiveness of interventions. The current findings do not to demonstrate a causality link between frequent internet gaming and multitasking performance. However, they may potentially provide useful information for researchers in choosing a sensitive task paradigm to evaluate the effect of intervention programs for internet gaming on an individual’s multitasking ability.

## Supporting information

S1 File Minimal data set for Tables 1–5. (XLSX)
